# Large retroperitoneal mass

**DOI:** 10.1016/j.sopen.2022.06.001

**Published:** 2022-06-20

**Authors:** Ryan Lamm, Wilbur B. Bowne

**Affiliations:** Department of Surgery, Thomas Jefferson University Hospital, Philadelphia, PA 19107

A 36-year-old woman with no significant medical history presented to the emergency department with rapid onset right flank abdominal pain. In the emergency department, workup revealed symptomatic anemia requiring 3 U of packed red blood cells. She received a computerized tomography scan which revealed a 22 × 15 × 14-cm fatty interpolar right-sided renal mass with numerous enlarged vessels and associated hemorrhage (Figure 1, *A* and *B*). The mass was embolized using multiple coils (Figure 1, *C* and *D*), and the biopsy taken revealed an admixture of thick dysmorphic blood vessels, smooth muscle, adipose tissue, and immunostaining for HMB-45 and melan-A (Figure 1, *E* and *F*). On 4-week follow-up, she was stable, with minimal pain, and was scheduled for elective resection. The large mass was removed via an open right subcostal incision. The specimen was removed en bloc with the right kidney, whereas the right adrenal gland was spared. The patient recovered without complication and was discharged home.

**Figure 1:**
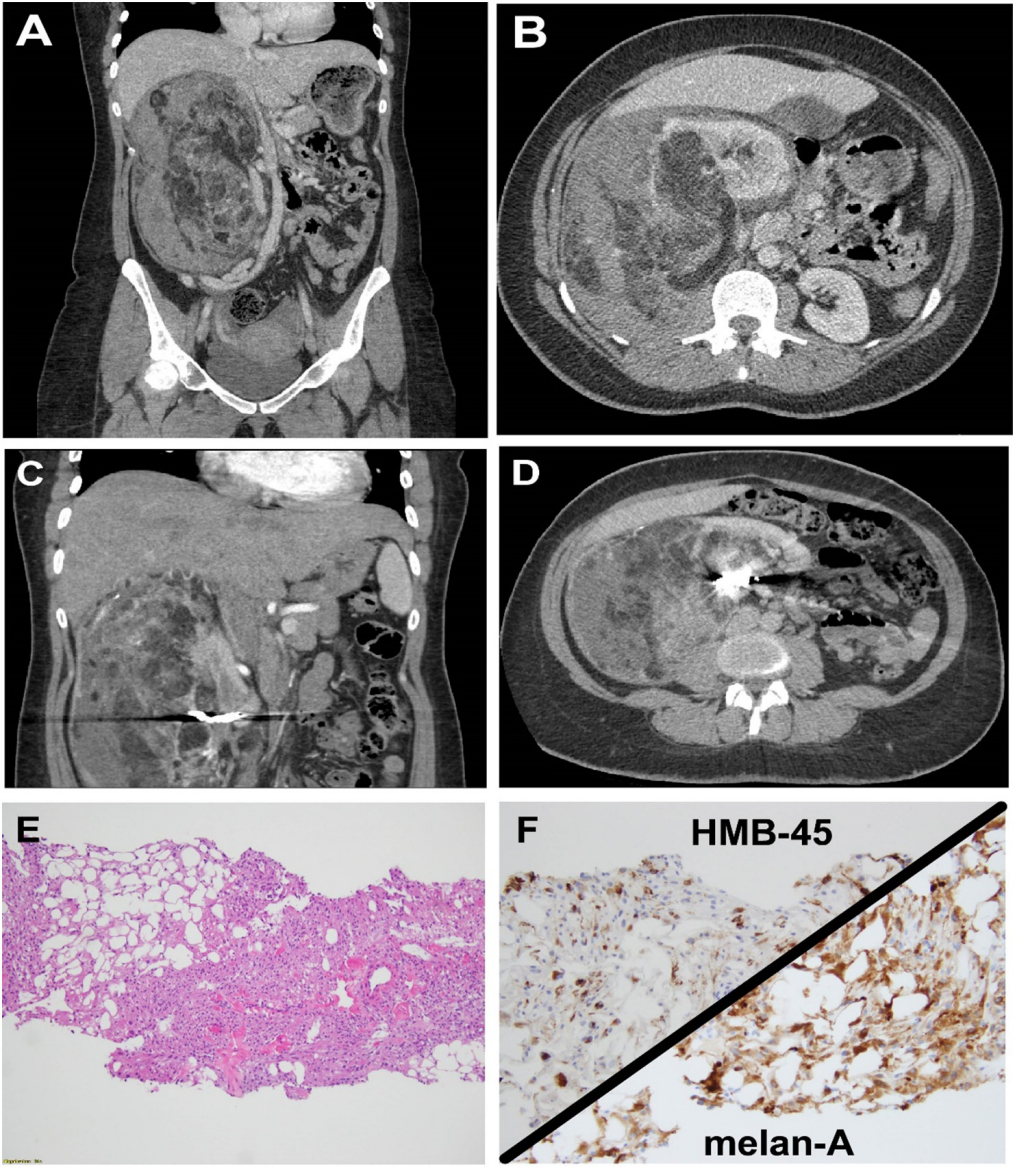
Large retroperitoneal mass.

Options:

A) Liposarcoma
B) **Angiomyolipoma**
C) Renal cell carcinoma
D) Renal abscess

## Author Contribution

RL and WB contributed to the manuscript writing and creation of the figure for this submission.

## Conflict of Interest

RL and WB have no conflicts of interest to disclose.

## Funding Source

RL and WB have no funding sources to disclose.

## Ethics Approval

Consent was provided by the patient for publication of the details of her case by RL and WB.

